# Time Balance and Family Functioning: The Role of Time Perspective in the Cohesion and Adaptability of Families with Adolescents

**DOI:** 10.3390/ejihpe14010008

**Published:** 2023-12-29

**Authors:** Cristián Oyanadel, Frank C. Worrell, Jorge Pinto-Vigueras, Sara Betancur, Tamara Véliz Tapia, Marisol Au-Castro, Génesis Peña-Reyes, Melissa González-Loyola, Wenceslao Peñate

**Affiliations:** 1 Department of Psychology, Universidad de Concepción, Concepcion 4030000, Chile; jpintov@udec.cl (J.P.-V.); saraebetancurs@gmail.com (S.B.); tveliz@udec.cl (T.V.T.); marisolaucastro@gmail.com (M.A.-C.); genesispena@udec.cl (G.P.-R.); meli_alejandra@hotmail.com (M.G.-L.); 2 Berkeley School of Education, University of California, Berkeley, CA 94720-1670, USA; frankc@berkeley.edu; 3 Department of Clinical Psychology, Psychobiology and Methodology, Universidad de La Laguna, 38200 La Laguna, Spain; wpenate@ull.edu.es

**Keywords:** time perspective, family functioning, balanced time profile, negative time profile, adolescents

## Abstract

Family functioning, understood as cohesion and adaptability, is critical in families with adolescent children, given the changes that this stage implies at the family level. Time perspective is one variable that can facilitate better family functioning through the way people give meaning to the process they live. In this study, we examined the relationship between family functioning and the time perspective of adolescent children’s parents. The FACES IV and ZTPI were administered to 276 parents of adolescents. Regression analyses indicated that the past positive, past negative, and future scores predicted family cohesion and adaptability, explaining at least 20% of the variance. Balanced families, with greater cohesion and adaptability, presented a higher level of past positive and future-oriented temporal perspectives, compared to unbalanced families, which presented a greater orientation to the past negative and deviated from the balanced temporal profile. The importance of considering the inter-relationship between family functioning and time perspective was discussed, considering its impact on the health and well-being of families with adolescents.

## 1. Introduction

Family functioning is defined as the ability of a family to meet the different needs it faces throughout the family life cycle [1] (Ortíz-Sánchez et al., 2023), and it is based on what [2] called the circumplex model of family systems. In this model, family functioning consists of three elements: family cohesion, referring to the degree to which family members interact or connect emotionally with each other; family adaptability, linked to the ability of a family to adapt to new changes as children progress through developmental stages; and family communication, understood as the communicative interaction that family members have with each other.

### 1.1. Family Functioning

To operationalize these elements, Olson (2014) [3] proposed two balanced and four unbalanced scales regarding family functioning. The two balanced scales are the cohesion scale, which assesses the emotional bond that family members have with each other, and the adaptability scale, which assesses the quality and ways in which leadership and organization are manifested in the family, in addition to how roles, rules, and negotiations are presented in relationships. The four unbalanced scales include (a) attachment, understood as connection and dependence between family members; (b) detachment, understood as significant disconnection and independence between members of a family system; (c) rigidity, understood as the presence of high control in relationships, with fixed roles and rules within the family; and (d) chaos, referring to the lack of leadership and order, with hasty decisions, and roles that are usually diffuse and interchangeable within the family and its relationships.

Family functioning has been studied in different age groups and has been related to different aspects of mental health. For example, family functioning has been found to be negatively related to depression [4,5,6], compulsive eating behaviors [7,8], and the quality of family attachment [9] and negatively related with the occurrence of suicidal ideation in adolescents [1,10,11,12]. In contrast, family functioning is positively related to medical treatment adherence [13], emotional expression [14,15], satisfaction and perceived well-being [16], and happiness [17]. Additionally, family functioning has been found to influence the appearance of behavioral problems in children [18,19], and, in addition, families with a member with autism spectrum disorder (ASD) present greater problems in their family functioning [20]. In contrast, family functioning is a relevant aspect to consider in order to improve the quality of life of caregivers [21].

The circumplex model of family functioning suggests that balanced families function better than unbalanced families [22,23]. Balanced families show low problematic functioning and are capable of facing stressful situations and managing tensions among their members, whereas unbalanced families report greater problems in terms of functioning, lack of strength, and protective factors [24]. Despite this general pattern, Crone and Fuligni (2020) [25] noted that it is normative for family functioning to fluctuate in certain life situations. For example, in the case of families with adolescents, family functioning fluctuates between balanced and unbalanced as children transition from childhood to adulthood.

Adolescence involves biological changes, such as physical changes or brain maturity [26], and psychological and social changes, which involve the incorporation of the adolescent into society through social responsibility, the establishment of personal goals, the fulfillment of cultural milestones (such as the formation of a couple), and the development of one’s own identity [27]. Therefore, parents must make family rules more flexible to promote independent decision making in their children, which allows them to prepare to live in society.

### 1.2. Family Functioning and Time Perspective

One aspect that may contribute to the understanding of functioning difficulties in families with adolescents is the time perspective, an unconscious process in which life experiences are attributed to time frames, which people use to make sense of their experiences [28]. The time perspective incorporates awareness of past and future events, which are influenced by cognitive (beliefs), emotional (affect associated with events), and social (cultural elements) aspects learned during the primary socialization process [29,30,31]. The time perspective tends to be considered as a stable element in human beings, as a personality trait, shaped by culture and interaction with the environment, such as religion, family, and personal values, generating a bias or inclination toward one of the time perspectives, which becomes the dominant perspective through which events are interpreted. For example, changes in the family cycle such as traumas, changes in religion, emigrations, or deaths, to name a few changes, impact the time perspective of family members, making them more future-oriented or more hopeless [29,31,32].

Zimbardo and Boyd (1999, 2009) [28,31] proposed five temporal dimensions: (a) past negative (PN), related to a negative attitude toward past events; (b) past positive (PP), which is related to a positive attitude toward past events; (c) present hedonistic (PH), defined as the orientation toward pleasure and enjoyment of what is experienced in the present; (d) present fatalistic (PF), defined as pessimism and hopelessness due to the events being experienced; and (e) future (F), understood as the attitude of projecting oneself and seeking future goals. Based on this model, the authors constructed the Zimbardo Time Perspective Inventory (ZTPI), which assesses these five temporal orientations.

ZTPI scores can be used to calculate a balanced temporal profile (BTP; [31]) and a negative temporal profile (NTP; [33]). A balanced temporal profile includes a high positive past score, a moderate score on the future and hedonistic present scales, and a low score on the negative past and fatalistic present scales [31,33], and this profile is considered optimal for well-being and health. A deviation from the balanced temporal profile (DBTP) can negatively affect health, well-being, affection, happiness, work success, and interpersonal relationships [30,31,34]. A negative temporal profile includes a low positive past score, a moderate score on the future and hedonistic present scales, and a high score on the negative past and fatalistic present scales [33]. Unlike with the DBTP, a deviation from the negative temporal profile (DNTP) is positively associated with perceived well-being as well as health [35].

Different time perspectives, as well as BTP and NTP, have been linked to different mental health variables. For example, perceived well-being has been positively associated with a positive past orientation, a future orientation, and a balanced time profile, whereas it has been negatively related to past negative and present fatalistic orientations [36,37,38,39,40]. Similarly, mental health and satisfaction with life have been positively related to a balanced time perspective [41,42,43,44]. It has also been observed how a present- and future-oriented perspective is positively related to higher self-esteem, whereas a greater orientation to the past is associated with lower levels of self-esteem [45]. In contrast, evidence has also demonstrated the mediating and predictive role of time perspective in emotional regulation [46], perceived well-being [47,48], perceived stress [49], decision making [50], depressive symptomatology [51], and suicide [52,53,54].

There is also evidence indicating that family characteristics and time perspective play an important role in the development of healthy and unhealthy behaviors in adolescents and emerging adults. Lin (2023) [55] linked time perspective with parenting and gratitude perceived by young adults. This researcher found that young adults who had high positive past and low negative past scores were more grateful to parents who were more affectionate, while young adults who were more oriented to the negative past were less grateful. Lin concluded that time perspective was a partial mediator of the association between parental care and gratitude. Stolarski et al. (2021) [56] studied how high family cohesion allowed adolescents to value, to a greater extent, the positive events that occurred throughout their lives, thus developing adaptive and healthy time perspectives, while adolescents in families with high family conflict developed less healthy temporal perspectives. In the case of the DBTP, the evidence indicates that students who report low acceptance within their family have higher DBTP scores compared to those who report higher levels of family acceptance, with the DBTP also being a moderator between school burnout and the development of depressive symptoms [57].

These studies notwithstanding, there is still limited evidence about the relationship between time perspective and family functioning. Specifically, cohesion and adaptability can be key in helping a family cope with its adolescent members and in the generation of healthy temporal perspectives [56]. Thus, time perspective together with family functioning can be relevant antecedents to consider in the development of more precise intervention strategies in the school and health contexts, in addition to the development of appropriate parental tools for this developmental period.

Therefore, the main objective of this research was to examine the association between family functioning and time perspective in parents of adolescent children. The specific objectives to be answered were how to (a) assess the relationships between family functioning and the temporal profiles of fathers and mothers of adolescent children; (b) evaluate the role of family cohesion in the time perspective of fathers and mothers of adolescent children; (c) examine the role of family adaptability in the time perspective of fathers and mothers of adolescent children; and (d) examine the role of time perspective variables in relation to the type of family functioning.

## 2. Materials and Methods

### 2.1. Design

A cross-sectional observational design was used, in which the dimensions of family cohesion and adaptability were quantitatively related to the different temporal perspectives postulated by Zimbardo and Boyd (1999) [28], which include negative past, past positive, present hedonistic, present fatalistic, and future. In addition, the relationship of family cohesion and adaptability to the deviation from the balanced and negative temporal profiles was assessed.

### 2.2. Participants

Using a non-probabilistic convenience sample, data were collected from 276 fathers and mothers of adolescent children attending school. The inclusion criteria were (a) being a father or mother and (b) having an adolescent child aged 14 to 17 years studying between the first and fourth years of high school who lived with their parents. La media de edad de los hijos fue de 15.42 años (SD = 1.15). Table 1 presents the sociodemographic characteristics of the sample, collected using a sociodemographic data sheet.

### 2.3. Instruments

Three evaluation instruments were used: a questionnaire to collect sociodemographic data, the Adaptability and Family Cohesion Evaluation Scale IV, and the Zimbardo Time Perspective Inventory. Sociodemographic data were collected at the beginning of the evaluation using a questionnaire. Questions were related to age, sex, marital status, and level of schooling of the participating fathers and mothers.

Family functioning was assessed with the Family Adaptability and Cohesion Assessment Scale IV (FACES IV, [58]). This instrument consists of 42 items that are answered on a Likert-type scale from 1 to 5, where 1 means completely disagree and 5 means completely agree [59]. As previously mentioned, FACES IV has six subscales that evaluate family functioning: two scales assessing balanced functioning (cohesion and adaptability) and four scales assessing unbalanced functioning (attachment, detachment, rigidity, and chaos; [3]). Only the balanced subscales were used in this study. Since scores on FACES IV have not been validated for the Chilean population, a Spanish version by Martínez-Pampliega et al. (2017) [59], adapted through cognitive interviews with parents or caregivers of Chilean adolescents, was used. In this study, the internal consistency was estimated with McDonald’s Omega coefficient, according to Kalkbrenner’s (2023) [60] suggestions, which were 0.72 for cohesion scores and 0.78 for adaptability scores.

Time perspective was measured using the ZTPI [28]. This scale is made up of 56 items divided into five subscales—past positive, past negative, present hedonistic, present fatalistic, and future—that assess how characteristic a statement is for the person, on a 5-point Likert-type scale, with 1 representing strongly disagree and 5 representing strongly agree. In the Chilean validation study, Cronbach’s alpha reliability estimates for the subscales scores were as follows: α = 0.59 for past positive, α = 0.80 for past negative, α = 0.79 for present hedonistic, α = 0.74 for present fatalistic, and α = 0.80 for future [61]. In this study, using McDonald’s omega, reliability estimates for ZPTI scores were as follows: ω = 0.79 for negative past, ω = 0.60 for positive past, ω = 0.78 for hedonistic present, ω = 0.66 for fatalistic present, and ω = 0.65 for future.

### 2.4. Procedure

Prior to data collection, cognitive interviews were conducted with 10 fathers or mothers of adolescent children, given that the FACES IV is not validated in Chile. A panel of experts suggested modifications, including precision of the prompt toward the nuclear family and replacement of some words with synonyms better known to the target population. Subsequently, the mothers and fathers of adolescents who were the study’s participants were informed in parent meetings about the objectives of the research and signed an informed consent. The data collection process lasted about 8 weeks. The collection strategy was through access via the schools to the parents’ meetings where the instruments were answered.

### 2.5. Data Analysis

For data analysis, the DBTP [48] and DNTP [33] coefficients were calculated. The DBTP was calculated according to the following formula:

$$\mathrm{DBTP}=\sqrt{(oPN-ePN)2+(oPP-ePP)2+(oPF-eFP)2+(oPH-ePH)2+(oF-eF)2}$$

in which (o) corresponds to the optimal score, and (e) corresponds to the score observed in the subjects. The optimal values for the five time perspectives are oPN = 1.95; oPP = 4.60; oPF= 1.50; oPH = 3.90; and oF = 4.00. In the case of the DNTP, the proposed formula is

$$\mathrm{DNTP}=\sqrt{(nPN-ePN)2+(nPP-ePP)2+(nPF-ePF)2+(nPH-ePH)2+(nF-eF)2}$$

in which (n) corresponds to the expected score, while (e) corresponds to the score observed in the subjects. The optimal scores in this case are nPN = 4.35; nPP = 2.80; nPF = 3.30; nPH = 2.65; and nF = 2.75. In both calculations, a result closer to 0 would indicate greater proximity to the balanced time profile or the negative time profile.

The Kolmogorov–Smirnov test was applied to determine the normality of the sample for family cohesion and adaptability, as well as the five time perspectives, the DBTP, and the DNTP. Since the data were not normally distributed, the Spearman correlation test was used to relate the family functioning variables to each of the time perspective variables, along with their deviations. Then, two stepwise multiple regressions were carried out, one with cohesion and the other with adaptability, with the objective of finding a model that would explain the predictive capacity of the different time perspective variables in the two main dimensions of family functioning.

Subsequently, using the percentile score conversion tables in the FACES IV manual [58], the level of each family was calculated based on its cohesion and adaptability. These analyses yielded three types of families for family cohesion—(a) very connected families, (b) connected families, and (c) somewhat connected families—and three types for family adaptability—(a) very adaptable families, (b) adaptable families, and (c) somewhat adaptable families. Once the levels were defined, the Kruskal–Wallis H test was used to compare the five temporal perspectives (positive past, negative past, hedonistic present, fatalistic present, and future) and the DBTP and DNTP of the sample with the different family types, based on cohesion and adaptability as independent variables. Additionally, the Bonferroni post hoc test was used to identify differences between families for family cohesion and adaptability.

Finally, integrating both dimensions of family functioning, the scores from the conversion table used for the previous analysis were used to form four family *typologies*: (a) dysfunctional, for families low in cohesion and adaptability; (b) adapted, for families with a high score on adaptability and a low score on cohesion; (c) cohesive, for families with a high score on cohesion but a low score on adaptability; (d) balanced, for families with high scores on cohesion and adaptability. The Kruskal–Wallis H with Bonferroni post hoc test was used to compare the four types of resulting families with the five dimensions of time perspective, together with the DBTP and DNTP. Cohen’s *d* was calculated for all group differences. All analyses were performed using the Statistical Package for the Social Sciences (SPSS) V.27.

## 3. Results

### 3.1. Correlations and Regressions

First, we examined the relationships between the levels of family cohesion and adaptability and the time perspective variables. Using the Spearman correlation test and a Bonferroni adjusted critical value of 0.005, the results indicated modest-to-moderate, positive, and statistically significant associations between cohesion scores and positive past, future, and DNTP scores and modest negative and statistically significant associations between cohesion scores and negative past, fatalistic present, and DBTP scores (see Table 2). Adaptability scores had modest-to-moderate, positive, and statistically significant relationships with positive past, future, and DNTP scores and a modest negative, statistically significant association with negative past scores.

Next, two stepwise multiple regression analyses were run to determine if the time perspective variables predicted family cohesion and adaptability. As the DBTP and DNTP are scores derived from the five ZPTI subscale scores, only the five subscale scores—positive past, negative past, hedonistic present, fatalistic present, and future—were used in these analyses. The results are summarized in Table 3 (predicting cohesion) and Table 4 (predicting adaptability).

The equation predicting cohesion was statistically significant: *F*(3, 272) = 34.40, *p* < 0.001, and *R*^2^ = 0.28. As can be seen in Table 3, three predictors were retained in the equation predicting cohesion. Past positive and future scores were positive predictors with interpretable effect sizes (i.e., β > 0.20; [62]), and past negative scores were a negative predictor with a small effect size. The equation predicting adaptability was also statistically significant (see Table 4): *F*(3, 272) = 22.47, *p* < 0.001, and *R*^2^ = 0.20. The same three predictors were retained in this equation with associations in the same direction, but only future had an interpretable effect size.

### 3.2. Differences among Family Types

#### 3.2.1. Family Types Based on Cohesion

The first set of family types were based on cohesion scores using the percentiles established by Olson et al. (2006) [58]. For family types based on cohesion, 60.5% of the sample (*n* = 167) were categorized as very connected families, 27.9% (*n* = 77) as connected families, and 11.6% (*n* = 32) as somewhat connected families. The three types of connected families significantly differed on positive past, *H*(2) = 30.267, *p* ≤ 0.001; negative past, *H*(2) = 8.254, *p* = 0.016; and future, *H*(2) = 26.89, *p* ≤ 0.001 (see Table 5). The interpretation of group differences was based on effect sizes.

The results indicated that very connected families had meaningfully (i.e., *d* > 0.40; [62]) higher positive past scores than connected families (*p* = 0.001, *d* = 0.51) and somewhat connected families (*p* ≤ 0.001, *d* = 1.10), and connected families had meaningfully higher positive past scores than somewhat connected families (*p* = 0.007, *d* = 0.63). In contrast, somewhat connected families had meaningfully higher negative past scores compared to very connected families (*p* = 0.011, *d* = −0.55) and connected families (*p* = 0.058, *d* = −0.56). Lastly, very connected families had meaningfully higher future scores than connected families (*p* = 0.001, *d* = 0.54) and somewhat connected families (*p* ≤ 0.001, *d* = 0.96). Very connected and connected families did not meaningfully differ on negative past scores (*d* = −0.07), and the difference between connected families and somewhat connected families fell just short of a meaningful difference on future scores (*d* = 0.39).

Statistically significant differences were also found in the DBTP scores among the three types of connected families, *H*(2) = 28.536, *p* ≤ 0.001. Post hoc analyses indicated that somewhat connected families deviated more from the balanced temporal profile than connected families (*p* = 0.003, *d* = −0.95) and very connected families *(p* ≤ 0.001, *d* = −1.22). The difference in DBTP scores between connected families and very connected families was also statistically significant (*p* = 0.001) and just short of meaningful (*d* = 0.40; Ferguson, 2009). With regard to DNTP scores, statistically significant differences were also found between families according to their level of family cohesion, *H*(2) = 29.516, *p* ≤ 0.001, and all three differences were statistically significant and meaningful: very connected families and connected families (*p* = 0.003, *d* = 0.46); very connected families and somewhat connected families (*p* ≤ 0.001, *d* = 1.00); and connected families and somewhat connected families (*p* = 0.021, *d* = 0.61).

#### 3.2.2. Family Types Based on Adaptability

With regard to family adaptability, 78.3% (*n* = 216) were categorized as very adaptable families, 19.5% (*n* = 54) as adaptable families, and only 2.2% (*n* = 6) as somewhat adaptable families [58]. Given the low frequency of somewhat adaptable families, we combined this group with the adaptable family group (21.7% of the sample; *n* = 60). We used the Mann–Whitney U test to compare the two family adaptability groups, and statistically significant differences were found between very adaptable and adaptable families on past positive, past negative, and future scores, as well as on DBTP and DNTP scores (see Table 6).

Very adaptable families had higher past positive (*U* = 4949.5, *p* = 0.005, *d* = 0.47) and future (*U* = 3872.5, *p* ≤ 0.001, *d* = 0.71) scores than adaptable families, and adaptable families had higher past negative scores than very adaptable families (*U* = 5098, *p* = 0.011, *d* = −0.40). The adaptable families deviated significantly more from the balanced temporal profile than the very adaptable families (*U* = 4855, *p* = 0.003, *d* = 0.40), whereas the very adaptable families were found to deviate more from the negative temporal profile than adaptable families (*U* = 4030, *p* ≤ 0.001, *d* = 0.69).

#### 3.2.3. Family Types Based on Cohesion and Adaptability

The third set of family types were created using *both* the cohesion and adaptability scores. First, low cohesion and low adaptability groups were created for families scoring at the 33rd percentile or lower for each variable, and high cohesion and high adaptability groups were created for families scoring at the 67th percentile or higher. Then, four family types were formed: dysfunctional families (low in both cohesion and adaptability), adaptable families (low in cohesion and high in adaptability), cohesive families (high in cohesion and low in adaptability), and balanced families (high in both cohesion and adaptability). Table 7 presents the distribution of the sample in the four resulting family types.

The four family groups were compared on the five temporal perspective scales—that is, we included the two present-oriented scores as exploratory—and the two temporal perspective composites (i.e., DBTP and DNTP) using the Kruskal–Wallis test (see Table 8 for means and standard deviations). The indicated results presented statistically significant differences among family types on past positive (χ^2^(3) = 36.44, *p* < 0.001) and future scores (χ^2^(3) = 44.01, *p* < 0.001), as well as on the DBTP (χ^2^(3) = 24.16, *p* < 0.001) and DNTP composites (χ^2^(3) = 42.47, *p* < 0.001). Family types did not meet the criterion for a significant difference (*p* < 0.01) on past negative scores (χ^2^(3) = 8.97, *p* = 0.03) and did not significantly meet it on present hedonistic and present fatalistic scores. Nonetheless, given the small numbers of families classified as adaptable and cohesive, we used effect sizes to compare the families on the temporal perspective subscale scores and composites. In keeping with best practice, effect sizes (*d* ≥ 0.41; [62]) were used to examine differences in time perspective scores among family types.

**Figure 1:**
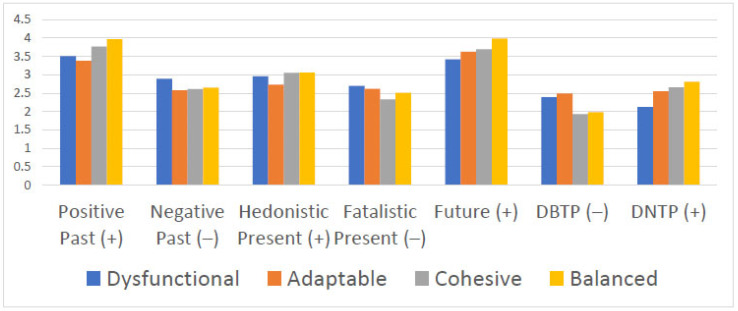
Patterns of time perspective scores by family type.

The differences in the pattern of results are presented in Figure 1. As can be seen in the figure, balanced families typically had higher scores than dysfunctional families on positive time constructs and lower scores on negative time constructs, with adaptable and cohesive families falling in the middle. The meaningful differences among the family types are presented in the next paragraph.

With regard to positive past, all comparisons between families yielded meaningful differences (0.43 < *d* < 1.15), except for one: dysfunctional families did meaningfully differ from adapted families (*d* = −0.24). The largest difference was in favor of balanced families relative to dysfunctional families (*d* = 0.97) and adaptable families (*d* = 1.15). On negative past, dysfunctional families reported meaningfully higher scores than adaptable (*d* = −0.59), cohesive (*d* = −0.54), and balanced (*d* = −0.41) families, but the latter three groups’ scores did not meaningfully differ (0.05 *< d* < 0.11; see Figure 1). Although the statistical analysis indicated no statistically significant differences for hedonistic present scores, three comparisons yielded meaningful differences. Adaptable families reported meaningfully lower scores than the other three family types: dysfunctional (*d* = −0.44), cohesive (*d* = −0.59), and balanced (*d* = −0.53). There was one meaningful difference on fatalistic present scores: cohesive families reported lower scores than dysfunctional families (*d* = −0.67). With regard to future scores, four of the six comparisons yielded meaningful differences (0.55 < *d* < 1.20), with scores increasing from dysfunctional to adaptable to cohesive to balanced families. Adaptable and cohesive families had similar future scores (*d* = 0.10), and adaptable families fell just short of a meaningful difference from dysfunctional families (*d* = 0.40).

Meaningful differences were also found for the two composite scores. For DBTP scores, cohesive and balanced families (*d* = 0.08) and dysfunctional and adaptable families (*d* = 0.16) did not meaningfully differ; however, the other four comparisons yielded meaningful differences (−0.83 < *d* < −0.71), with cohesive and balanced families reporting lower DBTP scores than dysfunctional and adaptable families. In contrast, dysfunctional families reported meaningfully lower DNTP scores than adaptable (*d* = 0.79), cohesive (*d* = 1.04), and balanced (*d* = 1.21) families, and adaptable families also reported lower DNTP scores than balanced families (*d* = 0.41). However, adaptable and cohesive families (*d* = 0.17) and cohesive and balanced families (*d* = 0.24) did not meaningfully differ.

## 4. Discussion

In this study, we analyzed the pattern of relationships between the two dimensions of family functioning (cohesion and adaptability), on the one hand, and five time perspective scores and two time perspective composite scores, on the other hand, in parents with adolescent children. The first objective was to assess the associations between family functioning and the temporal scores of the fathers and mothers of adolescent children. The results indicated that cohesion and adaptability had statistically significant positive associations with positive past, future, and DNTP scores and had statistically significant negative associations with negative past scores. Cohesion also had negative associations with fatalistic present and DBTP scores. However, not all associations were significant or interpretable based on effect size considerations, e.g., the temporal dimensions of the present. There were not statistically or practically significant associations between family functioning scores and hedonistic present, and adaptability was also not related to fatalistic present. These findings were supported with a stepwise multiple regression, with only positive past, negative past, and future being retained as predictors of cohesion and adaptability scores, accounting for 28% and 20% of the variance in these two variables, respectively. Positive past and future were meaningful predictors of cohesion, and future was a meaningful predictor of adaptability.

The second and third objectives were to determine the unique role of family cohesion and adaptability, respectively, in the time perspective of fathers and mothers of adolescent children. Using effect sizes, the results showed meaningful differences between both dimensions with past and future tenses and temporal deviations. Generally, families with greater cohesion and adaptability reported higher scores on positive past, future, and the DNTP, while families with lower cohesion and adaptability reported higher scores on negative past and the DBTP. Finally, the fourth objective was to examine time perspective scores in relation to family typologies based on both cohesion and adaptability. In these analyses, families classified as balanced reported higher positive past, future, and DNTP scores and lower negative past and DBTP scores than families classified as dysfunctional. Families classified as cohesive and adaptable were similar to balanced families on temporal scores (e.g., negative past) or fell between balanced and dysfunctional families (e.g., on future and DNTP scores).

### 4.1. Family Functioning and Temporal Perspectives

The results in the current study can be looked at in two ways: on the one hand, the prediction of the levels of family cohesion and adaptability by time perspective and, on the other hand, the family typologies that are related to positive, negative, and balanced time perspectives. The correlational analyses suggest that positive past and future are the strongest predictors of family cohesion, a point that is confirmed by the regression analyses that control for covariation among the predictors, with positive past carrying more weight. With regard to adaptability, both the correlational and regression analyses showcase the future as the strongest predictor, with positive past playing a lesser role. Thus, the perception of positive interactions in the past promotes family cohesion in the present with this connection, with expectations about the future contributing less to current satisfactory emotional engagement [36,55,56]. In the case of family adaptability, the greatest weight is carried by positive expectations of the future. In this sense, adaptability to contextual circumstances takes the future into account, which may be especially important for parents with adolescent children due to the need for adequate accommodation and assimilation of the changes that occur at this time [57,63].

With regard to predicting levels of family cohesion and adaptability, the valuation of the present does not play as significant a role. This finding could mean that the functioning of parents of adolescent boys and girls is more dependent on what has happened (and what will happen) than on the current circumstances. In this sense, parents’ distance themselves from the present situation (good or bad) and prefer to make value judgments based on how the family has been relating and how they have previously adapted [36] Although previous research indicates that present temporal dimensions are negatively associated with different life processes [64], the failure of a present orientation to contribute to family cohesion or adaptability can also be a negative indicator, to the extent that it can mean a distancing from the immediate reality in favor of past and future events that cannot be managed in the same way [47]. Future research could determine the plausibility of these conjectures.

The contrasts among the four created family typologies also reveal contrasting patterns, with balanced functioning (i.e., high levels of cohesion and adaptability) being associated with more positive time perspectives: higher levels of positive past and future and a larger deviation from the negative profile, alongside a smaller deviation from the balanced profile. Additionally, three of the four family types—that is, adaptable, cohesive, and balanced—do not differ in their valuation of negative past, with the three groups reporting lower scores than dysfunctional families. The present time perspective scores also play a less important role in differentiating among the four groups. For example, with the exception of dysfunctional families reporting higher scores than cohesive families, the four family types do not differ in their scores on fatalistic present. Similarly, adaptable families report lower hedonistic present scores than the other three groups. Thus, the negative past and the present time perspectives may be less important to family functioning than the positive past and future time constructs.

Generally speaking, cohesive families are more similar to balanced families than adaptable families, who are more similar to dysfunctional families than the cohesive group. In analyzing the differences among the typologies, cohesive families differ from balanced families only by slightly lower positive past and future scores. However, adaptable families (high in adaptability and low in cohesion) have substantially lower scores than balanced families on both positive past and future as well as lower hedonistic present scores. This finding is supportive of the idea that it is the level of cohesion that has the greatest weight in producing the association between a balanced time perspective and good family functioning. Considering cohesion as the emotional bond for the family [3], previous results [46,64], show that the relationship between emotional regulation/dysregulation is directly associated with time perspective. In contrast, family functioning is also associated with better resource management to control emotional disturbances [6,12,15]. The connection between good family functioning and a balanced time perspective makes sense, at least in that both are conditions that favor better regulation of emotions, both in normal conditions and in psychopathological conditions (especially anxiety and depression). The mechanisms by which this connection is established remain to be elucidated.

### 4.2. Limitations and Implications

Among the main limitations of this study, the Family Adaptability and Cohesion Evaluation Scale IV (FACES IV) does not have validity studies in Chile, so the subscales were adapted for use in the Chilean population using cognitive interviews, which were specifically carried out for this study. In addition, the sample size was limited, and it was a convenience sample of parents available to participate in this study. Furthermore, the entire sample corresponded to the Chilean population and its cultural framework. Therefore, the generalization of the results obtained for the population should be observed considering both limitations. In contrast, the ZTPI positive past scale does not show good reliability, which shows a difficulty that can be remedied or complemented with the use of robust instruments such as the Adolescent and Adult Time Inventory-Time Attitudes (AATI-TA) for the measurement of these variables [65]. The parents attended parent meetings at school, which may make the sample biased toward families that are more committed to formal aspects of their children’s education, and, therefore, are better functioning. However, although a larger sample is required for the results to be more robust, the current findings provide preliminary indications in the Chilean context about the relationship between time perspective and family functioning.

In future studies, it would be interesting to compare these findings with data on family functioning and time perspective in families at other stages of the family life cycle, especially families with pre-adolescent children and families with children who are already in emerging adulthood. The use of mixed methods might also provide a better understanding of the processes between time perspective and family functioning. Studies with family dyads would be especially interesting for a more complete understanding of this relationship. Nonetheless, the results of this study suggest that the time perspective of parents of adolescent children predicts the perceived closeness between family members, as well as their ability to adapt to the passage of time. On a practical level, these findings may be useful for parent education or in interventions or family guidance processes in adolescence, including as components cohesion, the balanced temporal perspective, and the role in emotion regulation. In sum, the results support the idea of promoting a positive and balanced time perspective in families with adolescents.

## 5. Conclusions

In this study, we examined the relationship between family functioning, understood as family cohesion and adaptability, and time perspective, understood as orientation toward the past, present, and future. The obtained results indicated how past and future time perspectives predict the level at which fathers and mothers of adolescent children perceive their ties as a family and the ability to adapt to the different evolutionary changes in the life cycle. Families that are balanced in their functioning were also more balanced in their time perspective. From this point of view, taking time perspective in the functioning of families with adolescent children into consideration is relevant, due to the implications for the health and well-being of adolescents and their family group.

## Figures and Tables

**Table 1:** Sociodemographic characteristics of the sample.

Variable	N	%
Caregiver		
Father	42	15.2
Mother	234	84.8
Total	276	100
Civil Status		
Single	47	17
Married	165	59.8
Widow	16	5.8
Divorced	20	7.2
Separated	21	7.6
Civil Union	5	1.8
Other	2	0.7
Total	276	100
Education Level		
Primary	16	5.8
Secondary	92	33.3
Technical	104	37.7
University	60	21.7
Other	4	1.5
Total	276	100
Child High School Level		
1st year	109	39.5
2nd year	75	27.2
3rd year	72	26.1
4th year	15	5.4
Other	5	1.8
Total	276	100

**Table 2:** Correlations between the balanced dimensions of the FACES IV and the ZTPI, DBTP, and DNTP scales.

	Past Positive	Past Negative	Present Hedonistic	Present Fatalistic	Future	DBTP	DNTP
Cohesion	0.42 *	−0.18 *	0.11	−0.16 *	0.39 *	−0.35 *	0.41 *
Adaptability	0.28 *	−0.16 *	0.03	−0.07	0.43 *	−0.12	0.37 *

Note: FACES IV = Family Adaptability and Cohesion Evaluation Scale fourth version; ZTPI = Zimbardo Time Perspective Inventory; DBTP = Deviation from the Balanced Temporal Profile; DNTP = Deviation from the Negative Temporal Profile. * *p* < 0.005.

**Table 3:** Stepwise multiple regression analysis predicting cohesion levels by time perspective variables (N = 276).

Variable	Coefficient		t	*p*	95% C.I.	
	B	β				
Constant	14.46		6.50	<0.001	10.08	18.83
Past Positive	2.88	0.37	6.61	<0.001	2.02	3.74
Future	1.78	0.22	4.02	<0.001	1.01	2.66
Past Negative	−0.87	−0.13	−2.37	0.018	−1.60	−0.15

Note: C.I. = confidence interval.

**Table 4:** Stepwise multiple regression analysis predicting adaptability levels by time perspective variables (N = 276).

Variable	Coefficient		t	*p*	95% C.I.	
	B	β				
Constant	16.43		6.64	<0.001	11.61	21.39
Future	2.87	0.34	5.78	<0.001	1.89	3.84
Past Negative	−1.11	−0.15	−2.6	0.008	−1.92	−0.30
Past Positive	1.23	0.15	2.54	0.012	0.28	2.19

Note: C.I. = confidence interval.

**Table 5:** Descriptive statistics for family cohesion groups by temporal perspective.

Family Cohesion	Temporal Perspective				
	Past Positive **	Past Negative *	Future **	DBTP **	DNTP **
	M (SD)	M (SD)	M (SD)	M (SD)	M (SD)
Very Connected (*n* = 167)	3.83 (0.48)	2.69 (0.61)	3.83 (0.45)	2.02 (0.63)	2.61 (0.61)
Connected (*n* = 77)	3.59 (0.44)	2.73 (0.50)	3.58 (0.50)	2.26 (0.54)	2.34 (0.55)
Somewhat Connected (*n* = 32)	3.27 (0.64)	3.02 (0.55)	3.38 (0.55)	2.78 (0.57)	2.01 (0.52)

Note: DBTP = Deviation from the Balanced Time Profile; DNTP = Deviation from the Negative Time Profile. * *p* < 0.016. ** *p* < 0.001.

**Table 6:** Descriptive statistics for family adaptability groups by temporal perspective.

Family Adaptability	Temporal Perspective				
	Past Positive **	Past Negative *	Future **	DBTP **	DNTP **
	M (SD)	M (SD)	M (SD)	M (SD)	M (SD)
Very Adaptable *n* = 216	3.74 (0.52)	2.69 (0.58)	3.78 (0.48)	2.10 (0.59)	2.56 (0.6)
Adaptable *n* = 60	3.50 (0.50)	2.92 (0.54)	3.44 (0.49)	2.36 (0.61)	2.15 (0.56)

Note: DBTP = Deviation from the Balanced Temporal Profile; DNTP = Deviation from the Negative Temporal Profile. * *p* < 0.011. ** *p* < 0.005.

**Table 7:** Distribution of family types in the studied sample.

		Cohesion	
		Low	High
Adaptability	Low	Dysfunctional (*n* = 70)	Cohesive (*n* = 16)
	High	Adaptable (*n* = 20)	Balanced (*n* = 84)

**Table 8:** Time perspective scores by family type.

Family Type	Past Positive	Past Negative	Present Hedonistic	Present Fatalistic	Future	DBTP	DNTP
	M (SD)	M (SD)	M (SD)	M (SD)	M (SD)	M (SD)	M (SD)
Dysfunctional	3.51 (0.49)	2.89 (0.49)	2.96 (0.52)	2.70 (0.55)	3.42 (0.49)	2.39 (0.54)	2.12 (0.50)
Adaptable	3.38 (0.70)	2.58 (0.65)	2.73 (0.54)	2.62 (0.85)	3.63 (0.64)	2.49 (0.82)	2.55 (0.67)
Cohesive	3.77 (0.51)	2.61 (0.62)	3.05 (0.55)	2.33 (0.60)	3.69 (0.49)	1.93 (0.62)	2.66 (0.59)
Balanced	3.97 (0.46)	2.65 (0.66)	3.06 (0.64)	2.51 (0.64)	3.99 (0.46)	1.98 (0.61)	2.81 (0.62)

Note: DBTP = Deviation from the Balanced Temporal Profile; DNTP = Deviation from the Negative Temporal Profile.

## Data Availability

Please contact the corresponding author to get the data.
